# Longitudinal regimes of arts and cultural engagement and frailty among older adults in the United States: a g-formula approach

**DOI:** 10.1093/geronb/gbag080

**Published:** 2026-05-06

**Authors:** Feifei Bu, Martin Danka, Juanyi Zhu, Nina T Rogers, Jill K Sonke, Daisy Fancourt, Jessica K Bone

**Affiliations:** Research Department of Behavioural Science and Health, Institute of Epidemiology & Health Care, University College London, London, United Kingdom; Centre for Longitudinal Studies, UCL Social Research Institute, University College London, London, United Kingdom; Department of Senior Service Management, School of Big Health Management, Chongqing College of Mobile Communication, Chongqing, China; Department of Public Health, Environments and Society, Faculty of Public Health and Policy, London School of Hygiene and Tropical Medicine, London, United Kingdom; Center for Arts in Medicine, University of Florida, Gainesville, Florida, United States; Research Department of Behavioural Science and Health, Institute of Epidemiology & Health Care, University College London, London, United Kingdom; Research Department of Behavioural Science and Health, Institute of Epidemiology & Health Care, University College London, London, United Kingdom; (Social Sciences Section)

**Keywords:** Health behavior, Receptive, Participatory, Longitudinal exposure regime, Time-varying confounder

## Abstract

**Objectives:**

A growing body of evidence links arts and cultural engagement (ACEng) with various health outcomes, however, its longitudinal relationship with frailty as a multidimensional clinical syndrome remains underexplored. This study aims to investigate the dynamic nature of ACEng and its impact on frailty, addressing critical methodological challenges.

**Methods:**

We analyzed longitudinal data from 3,775 older adults (aged 50+) in the U.S. Health and Retirement Study (2005–2018). ACEng was measured as receptive cultural events (e.g., concerts, movies, museums) and two participatory activities, including (1) singing or playing a musical instrument (2) doing arts and crafts. Frailty was assessed using a 50-item frailty index covering nine health domains. Data were analyzed using the g-formula approach.

**Results:**

ACEng was highly dynamic, particularly for receptive activities. Late and sustained engagements across three ACEng activities were associated with lower levels of frailty at follow-up. However, most of these effects were sensitive to adjustments of time-varying frailty, except for sustained engagement in cultural events. This sustained exposure was associated with a 0.35-point lower frailty compared to the never exposed (95% confidence interval: −0.53 to −0.17, *p* < .001).

**Discussion:**

The relationship between ACEng and frailty is complex and shaped by dynamic, potentially bidirectional relationships. This underscores the importance of using longitudinal designs and causal methods to understand ACEng regimes and their health impacts, accounting for time-varying confounders. Future research should explore distinct active ingredients and underlying mechanisms between receptive and participatory activities and how they can be leveraged to mitigate frailty and other health outcomes.

Worldwide, the proportion and absolute number of older adults are increasing rapidly. In the United States, the population aged 65 years and older is projected to increase from 58 million in 2022 to 82 million by 2050, representing an increase from 17% to 23% of the total population (U.S. Census Bureau, 2024). Frailty, a multidimensional clinical syndrome reflecting cumulative deficits in health, including physiological, physical, cognitive, and mental functions, is arguably the most challenging expression of population aging (Clegg et al., 2013; Thillainadesan et al., 2020). Frailty is highly prevalent in older adults, which increases the risks of adverse outcomes, such as falls, disability, delirium, mortality, and so forth (Fried et al., 2001; Persico et al., 2018; Song et al., 2010). It is also associated with higher risks of hospitalization, longer hospital stays, and higher health and social care costs (Chang et al., 2018; Kwok et al., 2020; Nikolova et al., 2022), imposing a heavy economic burden on individuals and public expenditure (Hajek et al., 2018). Therefore, in the context of population aging, it is crucial to understand protective factors that mitigate frailty and could be targeted with cost-efficient interventions.

One candidate protective factor is arts and cultural engagement (ACEng). ACEng is increasingly recognized as a health behavior (Rodriguez et al., 2024), comprising diverse active ingredients (e.g., aesthetics, physical or sensory stimulation, social interaction, evocation of emotion) that are beneficial to health, activating complex biological, psychological, social and behavioral mechanisms that may influence frailty (Fancourt et al., 2021; Fancourt & Finn, 2019). ACEng includes a diverse spectrum of activities that can be grouped into two overarching categories: (1) receptive engagement that involves experiencing arts and culture (e.g., attending concerts, visiting galleries and museums) and (2) participatory engagement that consists of creative and productive engagement (e.g., playing instruments, dancing, doing arts and crafts) (Bone et al., 2024; Rena et al., 2023). Although there may be overlapping active ingredients and mechanisms, receptive engagement typically affects health through absorption, reflection, and sensory stimulation, whereas participatory engagement operates through active creation, bodily motions, and actions. These differences can lead to differential health benefits as shown in previous research (Cuypers et al., 2012; Story et al., 2021).

There is ever-growing evidence on the relationship between ACEng and specific health outcomes, such as physical functioning, cognitive health, and depressive symptoms (Fancourt & Finn, 2019; Mattle et al., 2020; Viola et al., 2024), many of which are considered elements of frailty. ACEng also plays a major role in the management and treatment of conditions (Fancourt & Finn, 2019). However, the relationship between ACEng and frailty as an integrative and multidimensional syndrome is not well understood. Of the limited existing research, two small-scale interventional studies reported improvements in frailty after dance interventions among older adults (Meng et al., 2020; Zhang et al., 2023). Another interventional study reported improvement in frailty following a weekly intervention of participatory art-based activities (e.g., painting, drawing, book binding, rolled paper) over 3 months (Beauchet et al., 2021). A longitudinal observational study, focusing exclusively on receptive engagement, reported that older adults who attended cultural events (e.g., theater, concert, cinema, and museums) more frequently had a reduced risk of becoming frail and a slower progression of frailty over a follow-up period of 10 years (Rogers & Fancourt, 2020).

While findings on frailty and specific health conditions show promise, current research remains constrained by several methodological challenges. First, existing research predominantly measures ACEng at a single time-point, typically captured at baseline. This is problematic as ACEng is not a static event but constitutes a dynamic process with fluctuating patterns, through which health impacts develop and accumulate over time. Baseline measures cannot account for the difference between sustained and transient engagement. For example, baseline non-participants may engage later, while participants may discontinue their engagement. Second, the relationship between ACEng and frailty is likely to be bidirectional. Frailty may limit ACEng due to functional/mobility constraints, reduced motivation, or other access barriers (Nilsson et al., 2015). Frailty may therefore function as a time-varying confounder affecting ACEng and subsequent levels of frailty. Relatedly, previous studies typically adjusted only for baseline confounders, many of which are time-varying in nature (e.g., socioeconomic position). Accounting for time-varying confounders is needed to mitigate dynamic sources of bias in longitudinal analyses.

This study aimed to address these gaps by investigating the relationship between ACEng and frailty in older adults across 13 years (2005–2018) using longitudinal data from the Health and Retirement Study (HRS). Specifically, it examined potential differences between receptive and participatory engagement in relation to frailty. The data were analyzed using the g-formula approach, which allowed us to estimate the effect of longitudinal patterns of ACEng on frailty while accounting for time-varying confounders. By addressing key methodological limitations in prior research, our study provided robust evidence on how these patterns relate to frailty in older adults.

## Method

### Data

We used data from the HRS, a nationally representative study of more than 20,000 adults aged 50 years and over and their partners living in the United States. The initial cohort was first interviewed in 1992 and followed up every two years, and the sample is refreshed with younger cohorts every six years. In the fall of 2001, HRS launched the Consumption and Activities Mail Survey (CAMS) to a random subsample of the HRS households interviewed in 2000. CAMS included questions about the amount of time spent on individual activities (including arts and cultural engagement) and the level and pattern of household expenditures. CAMS was designed as a panel study where participants were followed up biennially in the off-year between HRS core waves (see Figure S1 in Supplementary Material).

The analytical sample comprised respondents who (i) joined CAMS in at least one CAMS wave in 2001, 2003, or 2005; (ii) were born in 1953 or earlier (consistent with the 2004 HRS age-eligible cohorts); and (iii) were alive at the end of follow-up when the outcome was measured (2018). There were 6,880 participants who joined CAMS at or before 2005. Among these participants, 2,838 (41.3%) died during the follow-up period and were excluded from the analysis, as frailty is undefined after death. Further 267 (6.6%) participants were excluded due to being born on or after 1954. This left an analytical sample of 3,775 (Figure S2).

### Measurement

Frailty was measured using a multidimensional frailty index of accumulated deficits adapted from Rogers et al. (Rogers et al., 2017), including 50 items within nine health domains: mobility, activities of daily living (ADL), instrumental activities of daily living (IADL), self-reported general health, mental health (measured by the eight-item Center for Epidemiologic Studies Depression Scale, CES-D) (Turvey et al., 1999), cognitive function (measured by sum scores for word recall, serial 7’s and backwards counting from 20), eyesight, hearing, and clinically diagnosed chronic conditions (high blood pressure or hypertension, angina, heart attack, congestive heart failure, abnormal heart rhythm, diabetes or high blood sugar, stroke, chronic lung disease, arthritis, cancer, any emotional/nervous/psychiatric problems). Each of the diagnosed chronic conditions was given a score of 1. The other eight domains were all rescaled between 0 and 1, rather than using raw sum scores, to prevent any single domain from being disproportionately weighted due to its number of sub-items. Therefore, the total frailty index ranged from 0 to 19 (see Table S1 in Supplementary Material), with higher scores indicating greater frailty. This was used as an end-of-follow-up outcome taken from the HRS core survey in 2018, and a time-varying covariate measured repeatedly in even years between 2004 and 2016.

ACEng was measured by the following items, each representing a different type of activity modeled separately in the analyses: how many hours did you spend last month: (1) attending concerts, movies, or lectures, or visiting museums, (2) singing or playing a musical instrument, (3) doing arts and crafts projects, including knitting, embroidery, or painting? Responses were recorded in hours, but recoded into binary variables (any engagement vs. no engagement) considering their skewed distributions with excessive zeros (Figure S3). These variables were measured repeatedly across waves in odd years between 2005 and 2017.

**Figure 1:**
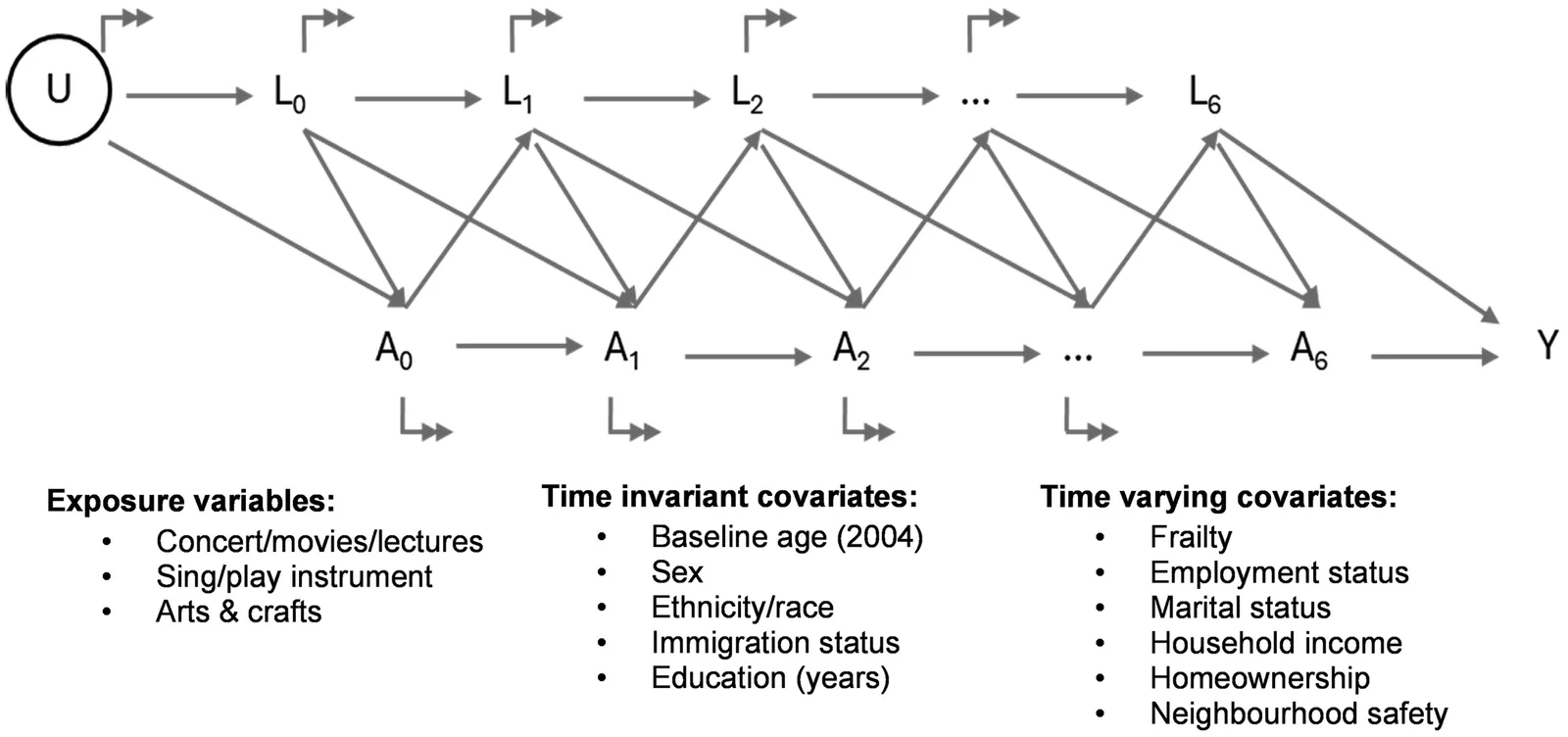
Causal diagram informing the analysis. Directed acyclic graph representing the assumed causal structure for estimating the effect of a time-varying exposure on an end-of-follow-up outcome. At each time point, the exposure (Aₜ) is influenced by the full history of prior confounders (Lₜ), its own past values, and unmeasured common causes (U). Each confounder (Lₜ) is affected by previous exposures, unmeasured factors, and its own past values. The outcome (Y) depends on the complete history of exposures and confounders. Although the DAG allows all past variables to influence all future variables, in practice these effects are expected to operate only up to some finite lag. The diagram explicitly shows arrows with a one-time-step lag between successive time points, while double-headed arcs originate from each variable and represent arrows extending from that variable to all future variables, summarizing longer-lag dependencies. DAG = Directed acyclic graph.

We considered a range of potential confounders based on the literature and substantive theory. The assumed causal structure was then represented using Directed Acyclic Graphs (DAGs, Figure 1). Time-invariant covariates measured at baseline (2004) comprised: age (years), sex (male, female), ethnicity (white, Black, Hispanic/other—including Alaskan Native, Asian, Native Hawaiian, and Pacific Islander), immigration status (born in the United States: yes, no), and education (school years). Time-varying covariates included: marital status (married, not married), employment status (employed, not employed), household income (in quartiles), home ownership (own, not own), and neighborhood safety (which could confound abilities to safely leave the home to participate in activities and maintain physical functioning; rated fair/poor, good, very good, excellent). These were measured repeatedly between 2004 and 2016. In our causal diagram, we allowed the full history of exposure and covariates to confound future relationships to avoid imposing arbitrary restrictions, although in practice, we expect their influence to extend only up to a certain time lag.

### Statistical analysis

Data were analyzed using the g-formula approach. This analytical approach is typically simulation-based: regression models are used to simulate how each person’s covariates and frailty would evolve over follow-up under a hypothetical engagement scenario, also known as a regime. To illustrate, one could simulate a person’s covariate profile and frailty under a regime in which they engaged in ACEng at every time point, and then under an alternative regime in which they did not engage at any time point. Repeating this process for all participants and averaging the simulated end-of-follow-up frailty values yields the mean frailty expected under each regime. These expected mean frailty levels can then be compared across regimes. In this simplified example, the estimated effect would be the difference in mean frailty between a regime in which everyone engaged in ACEng at every time point and a regime in which no one ever engaged. For a more detailed overview of the g-formula, see Naimi et al. (2017).

In our analyses, the hypothetical exposure regimes of interest were defined as: (1) early exposure: a scenario in which all participants would engage in ACEng during the initial three waves, followed by no ACEng in the final four waves, (2) late exposure: no ACEng in the first four waves, followed by engagement in the final three waves, (3) sustained exposure: ACEng across all waves. Each of these regimes was compared with a reference scenario (4), no exposure: no ACEng during follow-up. We started by estimating these contrasts without accounting for past frailty levels (Model I), followed by the inclusion of past frailty levels as a potential time-varying confounder to explore the possibility of reverse causation (Model II).

The main analysis was conducted using the parametric g-formula implemented using the multiple imputation (MI) estimator, gformulaMI. In this approach, counterfactual outcomes are simulated within a MI framework, even when data are fully observed. Effects and confidence intervals (CI) are then obtained using pooling rules for synthetic datasets. A key advantage is that the same framework can be naturally extended to handle missing data, allowing MI for missingness to be incorporated alongside effect estimation (Bartlett et al., 2025). Therefore, missing data were handled using MI under the missing-at-random assumption. MI for missingness included all variables that were also part of the substantive analysis as well as auxiliary variables, which included previously identified drivers of attrition such as past covariate levels, cognition, and ownership of a second home. The MI procedure used 50 imputed datasets and 40 iterations.

The standard implementation of g-formula requires the correct specification of regression models for the outcome and each time-varying covariate. We therefore conducted a sensitivity analysis to assess potential modeling misspecifications using the iterative conditional expectation (ICE) g-formula, an alternative semi-parametric approach that only requires specification of outcome models (Schomaker et al., 2019). We pooled the point estimates from the ICE estimator across the imputed datasets and compared them to those obtained from the gFormulaMI approach. We did not estimate CIs for the ICE approach, as this would require a computationally intensive bootstrap-MI procedure (Schomaker & Heumann, 2018). A detailed description of the analytical approach and assumptions for both the primary and sensitivity analyses is provided in Supplementary Methods S1. We additionally assessed the final frailty outcome models graphically using residual-versus-fitted and decile-based calibration plots. All analyses were conducted in R v4.4.1 using mice v3.17.0 (van Buuren & Groothuis-Oudshoorn, 2011), gFormulaMI v1.0.1 (Bartlett et al., 2025), and ltmle v1.3-0 (Lendle et al., 2017) packages.

## Results

### Descriptive statistics

Of the 3,775 participants in the analytical sample, 61.9% were female and 38.1% male (Table 1). Ethnic composition showed a slight overrepresentation of the white ethnic group (84.1%), relative to 11.7% Black, and 4.2% Hispanic or other ethnic minority groups. Over 90% of participants were U.S.-born. Mean education was 13.0 years (SD = 3.0), and mean baseline age was 63.8 years (SD = 7.9). At baseline, 71.4% of sample members were married, 45.4% were employed, and 86.8% were homeowners. There was an underrepresentation of people from the lowest income quartile (17.6%). Only 7.1% rated their neighborhood safety as fair or poor. The analytical sample was largely similar to the original sample, with some expected discrepancies due to restrictions related to mortality and age.

**Table 1:** Sample characteristics at baseline.

Variable	Response	Analytical sample (*n *= 3,775)			CAMS sample (*N *= 6,880)		
		*n*	Mean (SD)	**%**	*n*	Mean (SD)	**%**
**Baseline age**		3,775	63.8 (7.9)		6,880	67.6 (11.1)	
**Sex**	Male	1,438		38.1%	2,762		40.1%
	Female	2,337		61.9%	4,118		59.9%
**Ethnicity**	White/Caucasian	3,175		84.1%	5,819		84.6%
	Black/African American	441		11.7%	799		11.6%
	Other	159		4.2%	262		3.8%
**Born in the United States**	Yes	3,430		91.0%	6,290		91.6%
	No	340		9.0%	578		8.4%
**Education**	School years	3,775	13.0 (3.0)		6,875	12.5 (3.1)	
**Marital status**	Married	2,691		71.4%	4,463		65.3%
	Not married	1,078		28.6%	2,369		34.7%
**Employment status**	Employed	1,641		45.4%	2,192		34.5%
	Not employed	1,973		54.6%	4,167		65.5%
**Household income**	1^st^ quartile	637		17.6%	1,565		24.6%
	2^nd^ quartile	927		25.6%	1,811		28.5%
	3^rd^ quartile	1,045		28.9%	1,610		25.3%
	4^th^ quartile	1,008		27.9%	1,376		21.6%
**Home ownership**	Own	3,134		86.8%	5,177		81.6%
	Not own	477		13.2%	1,171		18.4%
**Neighborhood safety**	Fair/poor	256		7.1%	509		8.1%
	Good	718		19.9%	1,379		22.0%
	Very good	1,201		33.3%	2,103		33.5%
	Excellent	1,429		39.7%	2,291		36.5%

Table 2 shows the longitudinal transition patterns in ACEng. Among those who engaged in cultural events, 16.5% later discontinued, while 44.7% of initial non-engagers began engagement. For singing or playing instruments, only 9.3% of initial engagers discontinued, and 36.0% transitioned from non-engagement to engagement. Arts and crafts showed similar pattern with 9.9% transitioning from engagement to non-engagement, and 36.2% from non-engagement to engagement. Overall, all ACEng variables showed substantial changes over time, especially the transition from non-engagement to engagement. Notably, participatory activities demonstrated a greater stability, with fewer transitions from non-engagement to engagement (and vice versa) compared to receptive cultural events.

**Table 2:** Transitions over time for each of the ACEng variables.

Variable	Yes	No
**Cultural events**		
Yes	83.5%	16.5%
No	44.7%	55.3%
**Singing or instruments**		
Yes	90.7%	9.3%
No	36.0%	64.0%
**Arts and crafts**		
Yes	90.1%	9.9%
No	36.2%	63.8%

*Note*. ACEng = arts and cultural engagement.

### G-formula results

**Figure 2:**
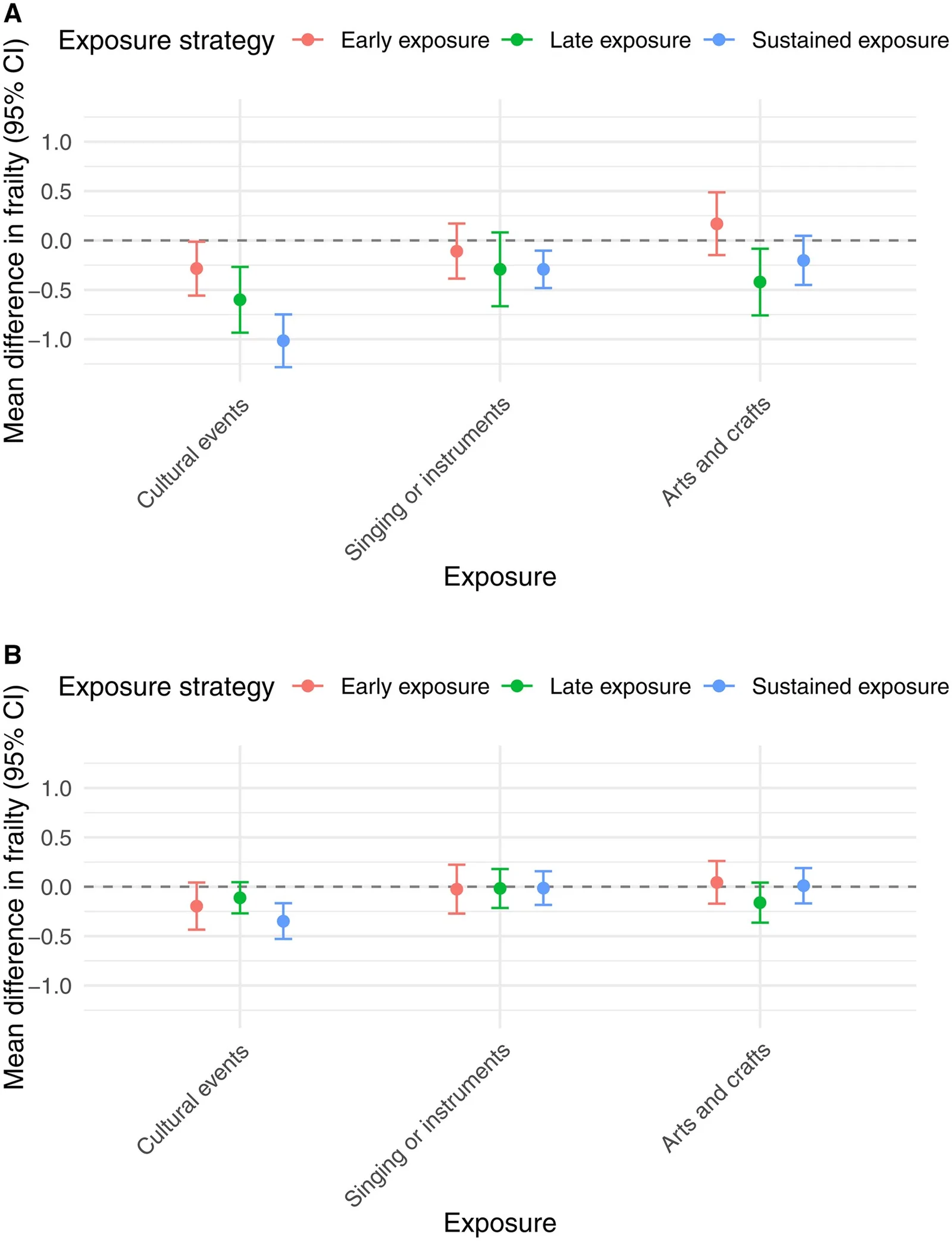
Estimated mean difference and 95% confidence intervals (reference group: no exposure) from the g-formula models: (A) Model I accounting for all covariates except for time-varying frailty; (B) Model II accounting for all covariates including time-varying frailty.

Figure 2 shows the estimated mean differences in frailty at the end of follow-up, with 95% CI, comparing hypothetical scenarios in which everyone followed a given exposure regime against no engagement at any time point. When frailty was not included as a time-varying confounder under Model I (Figure 2A), the estimated effects of cultural engagement were beneficial, with mean frailty levels lower than in the no-exposure scenario. Specifically, the estimated mean differences were −0.29 for early exposure (95% CI = [−0.56, −0.01], *p* = .041), −0.60 for late exposure (95% CI = [−0.93, −0.27], *p* < .001), and −1.02 for always exposed (95% CI = [−1.28, −0.75], *p* < .001). For singing or playing instruments, no evidence for a difference in mean frailty was found between the never and early or late exposure regimes. However, mean frailty was 0.29 points lower for always exposed (95% CI = [−0.48, −0.10], *p* = .004). Similarly, arts and crafts showed no frailty benefits for early exposure versus never exposed; while mean frailty was 0.42 points lower under the late exposure regime (95% CI = [−0.76, −0.08], *p* = .016), although we did not find clear evidence of a difference for sustained exposure (mean difference of −0.20 points, 95% CI = [−0.45, 0.05], *p* = .109).

After adjusting for time-varying frailty in Model II, most differences observed in Model I were substantially attenuated (Figure 2B). Specifically, neither singing or playing instruments nor arts and crafts showed any significant frailty benefits. For cultural events, while no difference was found for early or late exposure, sustained exposure resulted in an estimated benefit, with mean frailty lower by 0.35 points compared to never exposed ([−0.53, −0.17], *p* < .001). Sensitivity analyses using the ICE approach yielded nearly identical results (Table S2). Supplementary diagnostic plots for the final frailty outcome models did not indicate major model misspecification (Figures S4–S6).

## Discussion

By adopting a longitudinal approach to exposure, this study has demonstrated the dynamic nature of ACEng, particularly in receptive cultural events, where more changes over time are observed compared to participatory activities. After adjusting for frailty levels during the follow-up period, we have found little evidence that different ACEng regimes affect frailty at the end of follow-up, except for sustained engagement in receptive cultural events. This finding underscores the importance of exploring longitudinal engagement patterns and accounting for time-varying confounding by health-related outcomes in longitudinal studies of ACEng and health. Importantly, this is the first time that g methods have been applied to epidemiological analyses of ACEng, providing a substantial methodological advance within arts epidemiology.

Our study shows that there is substantial intra-individual variation in ACEng over time among older adults. This demonstrates the importance of accounting for the dynamic nature of ACEng to draw valid conclusions about its impact on health outcomes, such as frailty. In the existing literature, ACEng is commonly treated as a static measure (Fancourt & Steptoe, 2019; Jensen et al., 2024; Rena et al., 2023; Rogers & Fancourt, 2020). This approach, however, represents a methodological oversimplification without accounting for longitudinal changes in engagement as shown in our analyses, likely in response to changes in health status, social circumstances, motivation, time and resource availability (Baxter et al., 2022; Fancourt & Mak, 2020; Mak et al., 2023). This concern is particularly salient for older adults who are likely to experience these changes due to major life events such as retirement, widowhood, and the loss of family and friends, alongside health deteriorations. Existing longitudinal research on ACEng is limited and has largely relied on fixed-effects analytical approaches, focusing how changes in ACEng are related to changes in health (Fancourt et al., 2020; Finn et al., 2025; Wang et al., 2020). Our study extends the existing literature by providing new insights into the health implications of distinct longitudinal patterns of ACEng.

Prior to adjusting for time-varying frailty, but conditioning on all other covariates, our results show consistent benefits of late and/or sustained exposure across all three types of ACEng. In contrast, a benefit of early exposure is only observed for cultural events. Across different ACEng activities, cultural events have the largest effect sizes, showing a dose-response pattern from early exposure to late and sustained exposure. However, there is no evidence on cumulative benefits of participatory activities (singing or playing instruments, arts and crafts), with late and sustained exposure having similar effect sizes. Taken together, our findings suggest that there may be limited benefits of non-sustained early exposure to ACEng, reinforcing the importance of looking beyond baseline ACEng relative to future health outcomes, such as frailty. Further, our findings suggest distinct dynamic processes may underlie how receptive and participatory ACEng affect frailty. Through the lens of life course theory (Kuh et al., 2004), receptive engagement appears to operate through an accumulation of effects in older age, whereas participatory engagement shows a stronger recency effect. Future studies are needed to explore distinct active ingredients and underlying mechanisms that may be responsible for these differences.

These findings, however, should be interpreted with caution as most of the effects become negligible after accounting for time-varying frailty. This highlights the importance of addressing potential confounding by time-varying outcomes as prior states of frailty are likely to affect both present frailty and ACEng. Nevertheless, even after accounting for this, our analysis reveals a persistent effect of sustained exposure to cultural events on frailty at the end of follow-up, with lower frailty under sustained exposure. While this effect is modest, the finding is broadly consistent with previous studies showing that engagement in group-based cultural activities is associated with a lower risk of frailty onset (Fushiki et al., 2012) and cultural engagement overall can mitigate frailty progression (Rogers & Fancourt, 2020). The lack of benefit from early or late exposure further supports the explanation that receptive ACEng exerts its influence through an accumulative process over a prolonged period. This finding is in line with earlier work that links sustained cultural engagement with better self-reported health (Johansson et al., 2001) and wellbeing outcomes (Tymoszuk et al., 2020). The lack of evidence for participatory ACEng after adjusting for time-varying frailty is unexpected given the extensive evidence base linking these activities with specific health conditions (Fancourt & Finn, 2019). One potential explanation is the difference in settings and the associated active ingredients that shape the health benefits of ACEng. Specifically, receptive arts engagement is likely to involve leaving the home, acting as a vehicle to maintain social participation and, importantly, physical function. This contrasts with participatory activities examined in the present study, namely singing/playing instruments and doing arts and crafts, which can be done at home. Another potential explanation is that participatory ACEng operates through distinct pathways that are differentially associated with specific frailty indicators. Using a composite measure of frailty may have obscured these nuanced relationships. It is also possible that participatory ACEng is more susceptible to reverse causality. Specifically, the physical and functional limitations inherent in frailty may more directly constrain one’s ability to engage in participatory activities compared to receptive activities. Future work is needed to understand potential differences between receptive and participatory ACEng, how they change with increasing frailty, and their relationships with different health outcomes.

The main strengths of this study include using data from a large nationally representative panel study, using a causal inference framework to identify and address potential biases, and employing robust methods to deal with missing data. However, our study is not without limitations. First, ACEng is a broad concept, encompassing a wide range of activities, many of which are not measured in HRS, such as dancing that is shown to improve frailty in interventional research (Meng et al., 2020; Zhang et al., 2023). Moreover, due to their skewed distributions, ACEng measures are treated as binary in our analyses. Future research with more granular data may be better positioned to explore potential dose-response or threshold effects. Second, despite being derived from 50 items covering nine domains, our frailty measure is constrained by data availability and does not capture all potentially relevant domains. And as a self-reported measure, it is subject to reporting bias. Future studies incorporating richer clinical data on frailty are needed. Third, although we have adjusted for a range of time-invariant and time-varying confounders, informed by DAGs, we cannot rule out the possibility of residual confounding, which would undermine the sequential conditional exchangeability assumption. This is particularly important given that the remaining estimated effect after adjustment for time-varying frailty was modest in magnitude, and could therefore be sensitive to even relatively weak unmeasured confounding. Fourth, while our study examines ACEng over a 12-year period in mid-to-late adulthood, we acknowledge that the health benefits of ACEng may accumulate over the entire life course, or even intergenerationally. This calls for future research with even longer-term follow-ups. Finally, we recognize that gender is not a binary construct, although we had to treat it as such given the way data were collected. Further, as HRS combined a range of races/ethnicities into the Hispanic/other race/ethnicity category, we were not able to investigate the influence of specific race/ethnic identities, and associated racism and cultural caste systems. These limitations underscore the challenges of controlling for demographics and the need for improving methods for measuring and accounting for structural racism in research (Hardeman et al., 2022).

Overall, this study demonstrates the dynamic nature of ACEng, showing changes in engagement for a substantial proportion of older adults over a 13-year follow-up. Our findings suggest distinct temporal mechanisms for different types of ACEng, with receptive engagement showing evidence of effect accumulation and participatory engagement demonstrating greater susceptibility to confounding by prior frailty states. We call for future research that employs longitudinal designs and robust causal inference methods, moving beyond static baseline approaches, to capture the dynamic nature of ACEng and accurately quantify its effects on health. And more research is needed to disentangle the directionality of the relationship between ACEng and different health outcomes.

## Supplementary Material

gbag080_Supplementary_Data

## Data Availability

The raw HRS data are available from the RAND Center for the Study of Aging (https://hrsdata.isr.umich.edu/data-products/rand). The code used to perform analyses in this study is publicly available on GitHub at https://github.com/martindanka/hrs-frailty-gformulami.
