# Supplementary material for: Longitudinal regimes of arts and cultural engagement and frailty among older adults in the United States: a g-formula approach
Source: J Gerontol B Psychol Sci Soc Sci. 2026 May 6;81(7):gbag080. doi: 10.1093/geronb/gbag080 (PMC13257856; doi:10.1093/geronb/gbag080)
Supplement: gbag080_Supplementary_Data [file gbag080_supplementary_data.zip › HRS frailty-Supplement-R1.pdf]

Supplementary Materials

Longitudinal regimes of arts and cultural engagement and frailty among older adults in the United States: a g-formula approach

Contents

Supplementary Methods 2

Methods S1. Details on the g-formulaMI implementation 2

Supplementary Tables 4

Table S1 Measures of the multidimensional frailty index 4

Table S2 Results from the sensitivity analyses comparing to the main analyses (Model II) 5

Supplementary Figures 6

Figure S1 Study timeline 6

Figure S2 Sample selection diagram 7

Figure S3 Distribution of the arts and cultural engagement measures on the original scale (2005) 8

Figure S4 Diagnostic plots for the frailty outcome model with attendance at concerts/movies/lectures as the exposure. 9

Figure S5 Diagnostic plots for the frailty outcome model with singing or playing a musical instrument as the exposure. Panel (a) shows residuals versus fitted values for the final frailty outcome model........ 10

Figure S6 Diagnostic plots for the frailty outcome model with doing arts and crafts projects as the exposure. 11

References 12

Supplementary Methods

Methods S1. Details on the g-formulaMI implementation

Effect identification and assumptions

The identification of the causal effects of interest in our study relied on several untestable assumptions, including: (i) sequential conditional exchangeability, (ii) counterfactual consistency, (iii) no interference, and (iv) correct model specification.

Sequential conditional exchangeability assumption is a specific form of the no unmeasured confounding assumption, stating that at each timepoint, the potential outcome is independent of the exposure given the exposure and covariate history. We sought to make this assumption more plausible by adjusting for a wide range of time-varying confounders. However, as in most analyses of observational data, residual confounding cannot be ruled out.

Counterfactual consistency requires that the potential outcome under a specific exposure regime equals the observed outcome if that regime were realised. This implies that the exposure must be 'sufficiently well-defined'. We acknowledge that a strict interventionist interpretation of this assumption is unlikely to hold in the study, as our exposure encompasses various forms and intensities of cultural and arts engagement. That said, the assumption can be relaxed if the effect is viewed as a weighted average across exposure versions, implicitly weighted by their prevalence [1]. This may limit the transportability of our findings to other populations [2], thus future research should explore specific activity types/intensities and focus on cross-cultural comparisons.

No interference assumes that one individual's potential outcome is independent of others' exposure histories. We consider this plausible given participants' wide geographic distribution. For those in closer proximity, one person's engagement might influence another's frailty, but we reasoned that such effects would be small on average in the population and they would most likely operate through the individual's own engagement, thus we would expect minimal interference.

Correct model specification requires correctly specifying functional forms of relationships in statistical models. When using the parametric g-formula, models are required for the outcome as well as each time-varying covariate. Sensitivity analyses using the ICE approach suggested our findings were robust to misspecification in the covariate models. Nonetheless, our options for modelling the outcome were limited given the number of timepoints, so the model did not include interactions or non-linear terms. Yet, supplementary diagnostic plots did not suggest major misspecification of the final frailty outcome models (Fig S4-S6).

MI for missingness

We handled attrition and item nonresponse using multiple imputation (MI) with fully conditional specification, generating 50 imputed datasets with 40 iterations each. MI was performed on three occasions–once for each of the three ACEng exposure variables–excluding the other two exposure variables of interest.

Imputation models included all variables that were part of the substantive analysis. Additionally, we accounted for drivers of attrition, including variables used by the cohort team to derive the CAMS nonresponse weights. Because our analyses already included some of these variables or related constructs, auxiliary variables in the present study were limited to those obtained at or before CAMS 2001 and CAMS 2003, specifically:

- Past levels of time-varying covariates
- Cognition
- Difficulty managing money
- Ownership of a second home

All substantive variables were initially allowed to impute one another across time points. To limit model complexity, auxiliary variables measured at wave *t* were only used to impute variables at wave *t*+1.

The inspection of imputation diagnostics revealed some convergence issues, indicated by feedback loops in trace plots and potential scale reduction factors (PSRF) greater than 1.1 for several variables. These were resolved by modifying the imputation structure as follows: for frailty, housing tenure, and marital status at time *t*, we prevented each of these variables to be imputed by its own future values (*t*+1 onwards). For frailty specifically, we retained the final outcome value (frailty at the end of follow-up) as a predictor in all imputation models, while excluding frailty levels preceding CAMS 2001 and CAMS 2003.

To impute the variables, we used normal linear models for continuous variables that were part of the substantive analyses, logistic regression for binary variables, and ordinal logistic regression for ordered variables; continuous auxiliary variables were imputed using predictive mean matching.

GformulaMI for effect estimation

The MI for effect estimation also used 50 imputed datasets, giving a Monte Carlo sample size of $50 \times 3{,}775 = 188{,}750$. Auxiliary variables were not included in these substantive models. Each variable was simulated using the full history of treatment and covariates up to that timepoint. The models used to simulate the different variable types matched those described under MI for missingness.

Supplementary Tables

Table S1 Measures of the multidimensional frailty index

Domain	Question	Range
Mobility	Because of a health problem do you have any difficulty with walking several blocks?	0-1
	With walking one block	
	Climbing several flights of stairs without resting	
	Climbing one flight of stairs without resting	
	sitting for about two hours?	
	getting up from a chair after sitting for long periods?	
	stooping, kneeling, or crouching?	
	pulling or pushing large objects like a living room chair?	
	Reaching or extending arms above shoulder level	
	Lifting or carrying weights over 10 pounds, like a heavy bag	
	Picking up a 5p coin from a table	
ADL	Dressing, including putting on shoes and socks	0-1
	Walking across a room	
	Bathing or showering	
	Eating, such as cutting up your food	
	Getting in or out of bed	
	Using the toilet, including getting up or down	
IADL	Preparing a hot meal	0-1
	Shopping for groceries	
	Making telephone calls	
	Taking medication	
	Managing money, (e.g. paying bills and keeping track of expenses)	
General Health	Self-reported general health (fair/poor compared to excellent/very good/ good)	0-1
Mental health (CESD)	you felt depressed	0-1
	you felt that everything you did was an effort	
	Your sleep was restless	
	You were happy	
	You felt lonely	
	You enjoyed life	
	You felt sad	
	You could not get going	
Cognitive function	Immediate word recall	0-1
	Delayed word recall	
	Serial 7's subtraction: to subtract 7 from 100, and continue subtracting 7 from each subsequent number for a total of five trials	
	Backwards counting: to count backwards for 10 continuous numbers beginning with the number 20.	
Eyesight	Is your eyesight excellent, very good, good, fair, or poor using glasses or corrective lenses as usual?	0-1
Hearing	Is your hearing excellent, very good, good, fair, or poor [(using a hearing aid as usual)]?	0-1
Diagnosed chronic conditions	High blood pressure or hypertension	0-1
	Angina	0-1
	Heart attack (including myocardial infarction or coronary thrombosis)	0-1
	Congestive heart failure	0-1
	An abnormal heart rhythm	0-1
	Diabetes or high blood sugar	0-1
	A stroke (cerebral vascular disease)	0-1

Chronic lung disease such as chronic bronchitis or emphysema	0-1
Arthritis (including osteoarthritis, or rheumatism)	0-1
Cancer or a malignant tumour (excluding minor skin cancers)	0-1
Any emotional, nervous or psychiatric problems	0-1

Table S2 Results from the sensitivity analyses comparing to the main analyses (Model II)

Exposure	Regime	gformulaMI estimate	gformula ICE estimate	Difference
Cultural events	Ealy exposure	-0.196	-0.202	0.006
	Late exposure	-0.112	-0.117	0.005
	Always exposed	-0.348	-0.346	-0.002
Singing or instruments	Ealy exposure	-0.024	-0.056	0.032
	Late exposure	-0.018	0.013	-0.031
	Always exposed	-0.013	-0.031	0.018
Arts and crafts	Ealy exposure	0.044	0.050	-0.006
	Late exposure	-0.161	-0.162	0.001
	Always exposed	0.010	0.013	-0.003

Supplementary Figures

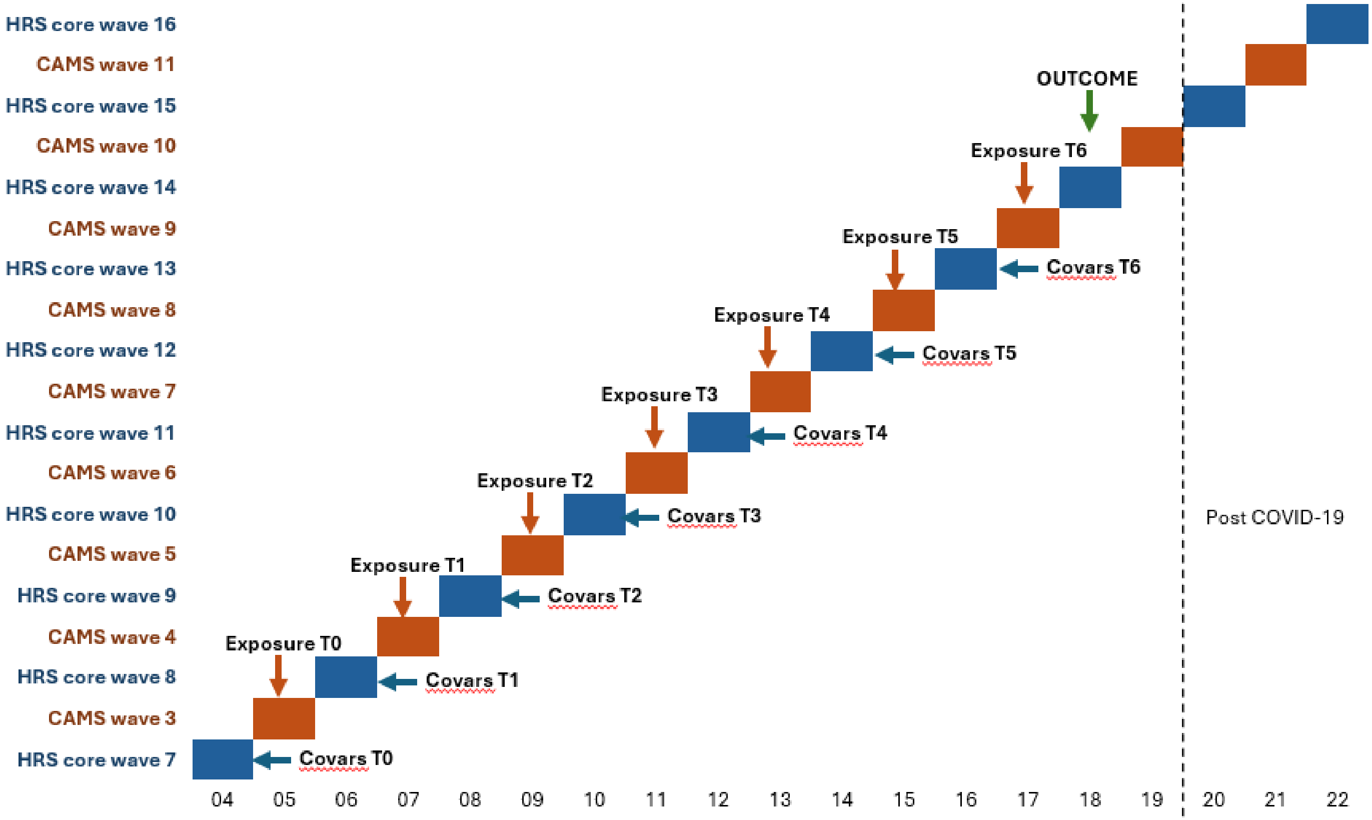

Figure S1 Study timeline

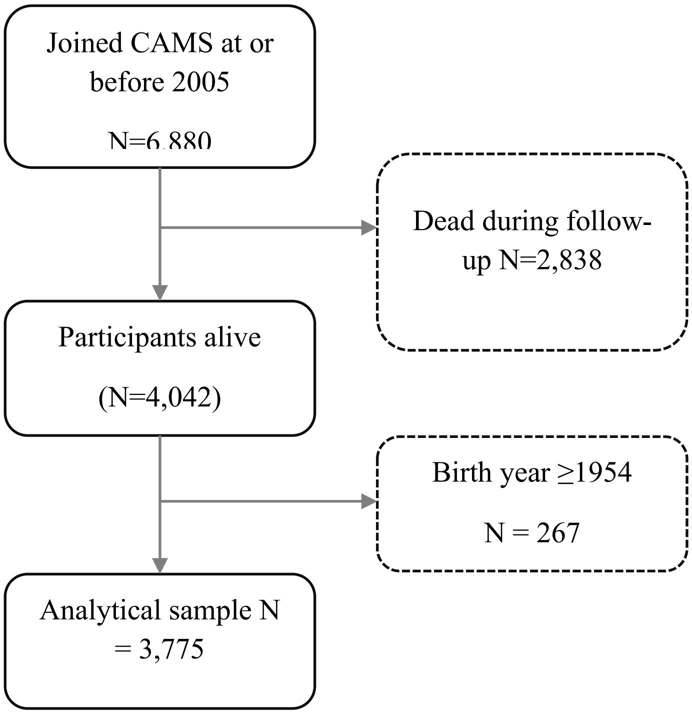

Figure S2 Sample selection diagram

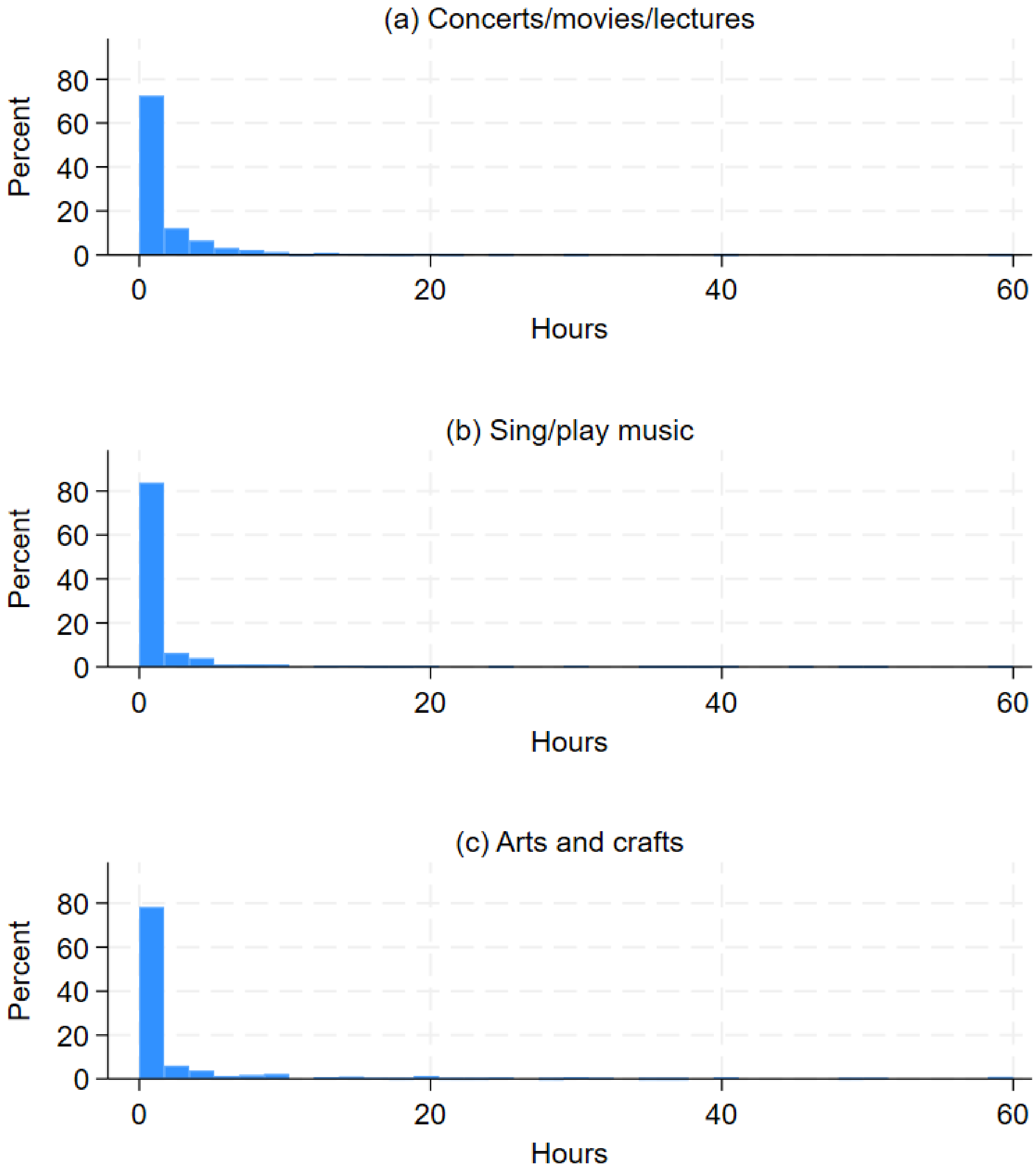

Figure S3 Distribution of the arts and cultural engagement measures on the original scale (2005)

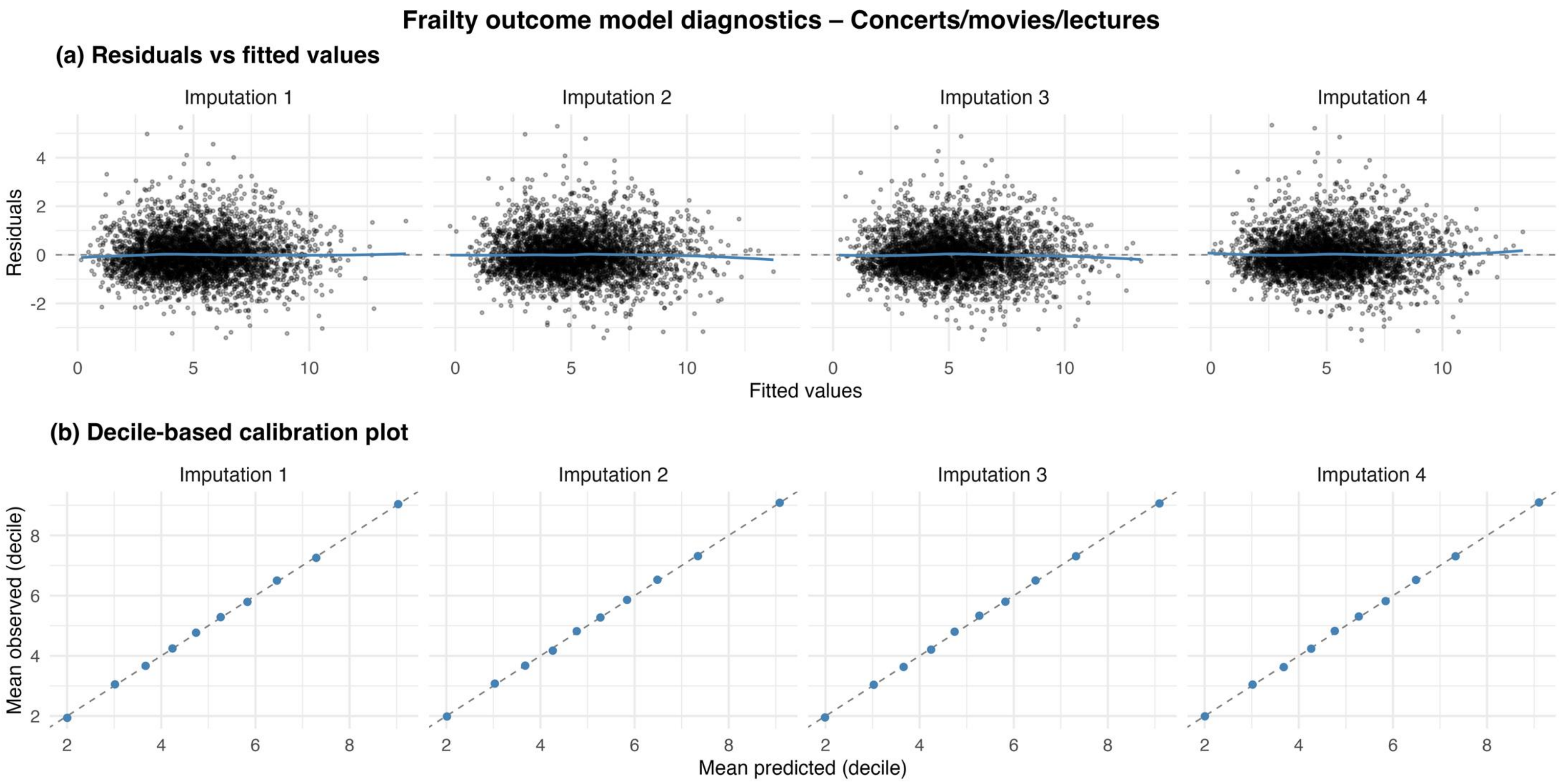

Figure S4 Diagnostic plots for the frailty outcome model with attendance at concerts/movies/lectures as the exposure. Panel (a) shows residuals versus fitted values for the final frailty outcome model. Panel (b) shows a decile-based calibration plot, with mean observed frailty plotted against mean predicted frailty within deciles of predicted values. The analysis involved two imputation steps: first, multiple imputation for missing data; second, a separate imputation-based procedure within gformulaMI for effect estimation. These diagnostics assess the substantive outcome model used for effect estimation, evaluated across datasets created by the first imputation step. The first four such datasets are shown for brevity, as patterns were similar across datasets.

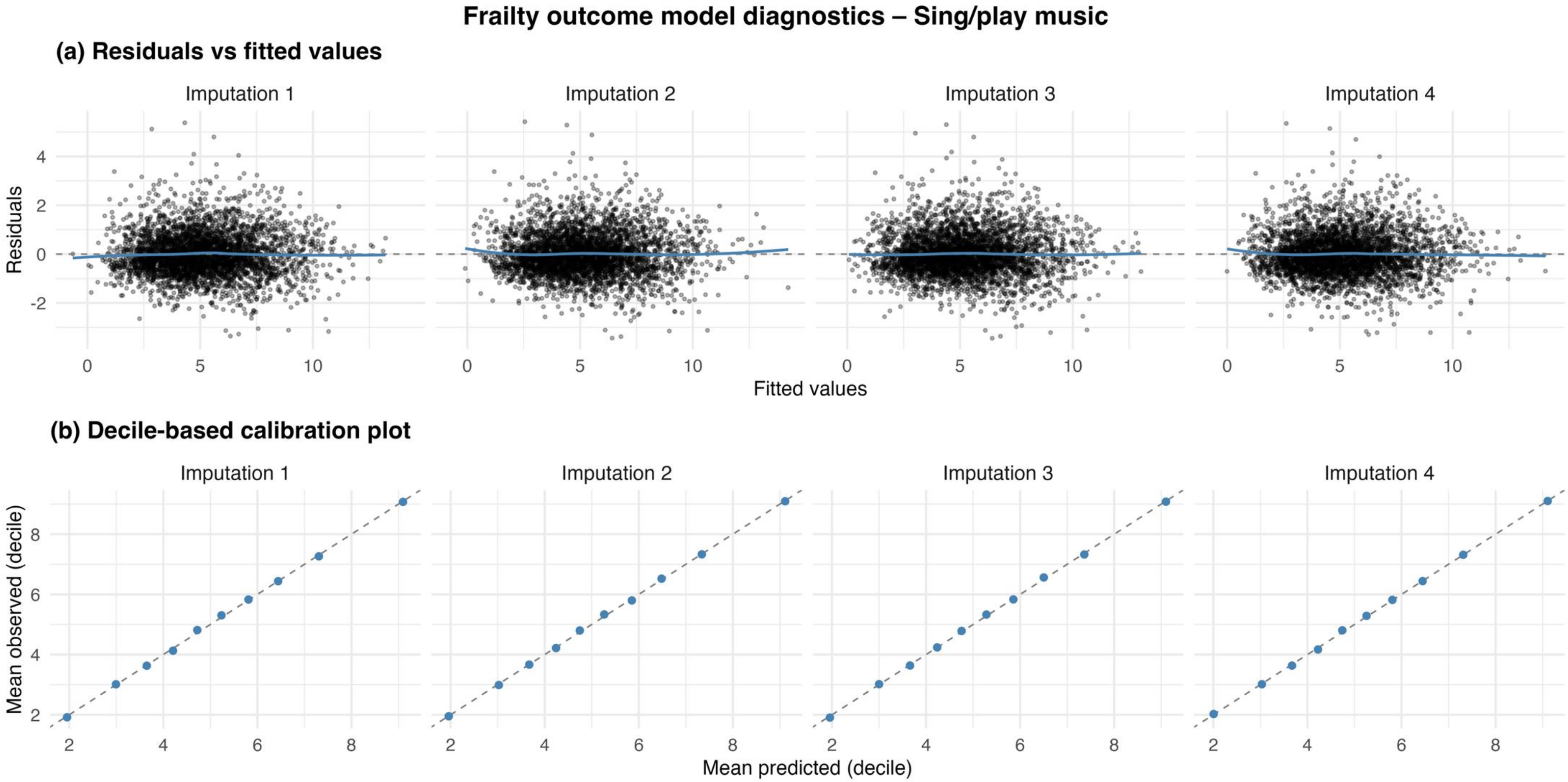

Figure S5 Diagnostic plots for the frailty outcome model with singing or playing a musical instrument as the exposure. Panel (a) shows residuals versus fitted values for the final frailty outcome model. Panel (b) shows a decile-based calibration plot, with mean observed frailty plotted against mean predicted frailty within deciles of predicted values. The analysis involved two imputation steps: first, multiple imputation for missing data; second, a separate imputation-based procedure within gformulaMI for effect estimation. These diagnostics assess the substantive outcome model used for effect estimation, evaluated across datasets created by the first imputation step. The first four such datasets are shown for brevity, as patterns were similar across datasets.

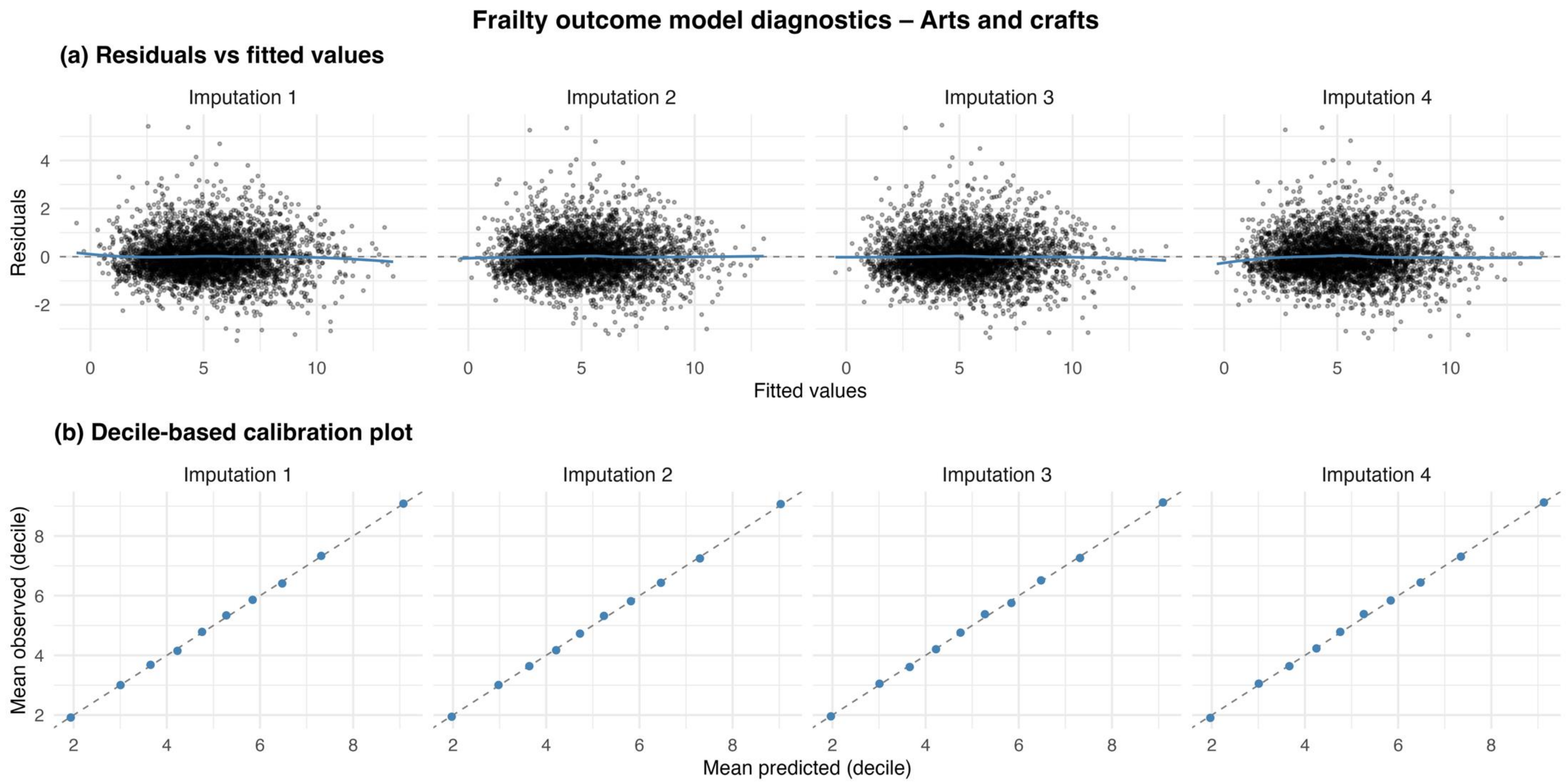

Figure S6 Diagnostic plots for the frailty outcome model with doing arts and crafts projects as the exposure. Panel (a) shows residuals versus fitted values for the final frailty outcome model. Panel (b) shows a decile-based calibration plot, with mean observed frailty plotted against mean predicted frailty within deciles of predicted values. The analysis involved two imputation steps: first, multiple imputation for missing data; second, a separate imputation-based procedure within gformulaMI for effect estimation. These diagnostics assess the substantive outcome model used for effect estimation, evaluated across datasets created by the first imputation step. The first four such datasets are shown for brevity, as patterns were similar across datasets.

References

[1] VanderWeele TJ, Hernan MA. Causal inference under multiple versions of treatment. J Causal Inference 2013;1:1–20. https://doi.org/10.1515/JCI-2012-0002.

[2] Hernán MA, Vanderweele TJ. Compound treatments and transportability of causal inference. Epidemiology 2011;22:368–77. https://doi.org/10.1097/EDE.0B013E3182109296.
